# Childhood Obesity and Respiratory Diseases: Which Link?

**DOI:** 10.3390/children8030177

**Published:** 2021-02-25

**Authors:** Emanuela di Palmo, Emanuele Filice, Alessandra Cavallo, Carlo Caffarelli, Giulio Maltoni, Angela Miniaci, Giampaolo Ricci, Andrea Pession

**Affiliations:** 1 Pediatric Unit, IRCCS Azienda Ospedaliero-Universitaria di Bologna, 40138 Bologna, Italy; emanuela.dipalmo@aosp.bo.it (E.d.P.); eman.filice@gmail.com (E.F.); alessandra.cavallo91@gmail.com (A.C.); Giulio.maltoni@aosp.bo.it (G.M.); angela.miniaci@aosp.bo.it (A.M.); giampaolo.ricci@unibo.it (G.R.); 2 Department of Medicine and Surgery, Pediatric Clinic, University of Parma, 43126 Parma, Italy; carlo.caffarelli@unipr.it

**Keywords:** obesity, asthma, airway dysanapsis, OSAS, leptin, adipose tissue, children

## Abstract

Prevalence of childhood obesity is progressively increasing, reaching worldwide levels of 5.6% in girls and of 7.8% in boys. Several evidences showed that obesity is a major preventable risk factor and disease modifier of some respiratory conditions such as asthma and Obstructive Sleep Apnea Syndrome (OSAS). Co-occurrence of asthma and obesity may be due to common pathogenetic factors including exposure to air pollutants and tobacco smoking, Western diet, and low Vitamin D levels. Lung growth and dysanapsis phenomenon in asthmatic obese children play a role in impaired respiratory function which appears to be different than in adults. Genes involved in both asthma and obesity have been identified, though a gene-by-environment interaction has not been properly investigated yet. The identification of modifiable environmental factors influencing gene expression through epigenetic mechanisms may change the natural history of both diseases. Another important pediatric respiratory condition associated with obesity is Sleep-Disordered Breathing (SDB), especially Obstructive Sleep Apnea Syndrome (OSAS). OSAS and obesity are linked by a bidirectional causality, where the effects of one affect the other. The factors most involved in the association between OSAS and obesity are oxidative stress, systemic inflammation, and gut microbiota. In OSAS pathogenesis, obesity’s role appears to be mainly due to mechanical factors leading to an increase of respiratory work at night-time. However, a causal link between obesity-related inflammatory state and OSAS pathogenesis still needs to be properly confirmed. To prevent obesity and its complications, family education and precocious lifestyle changes are critical. A healthy diet may lead to an improved quality of life in obese children suffering from respiratory diseases. The present review aimed to investigate the links between obesity, asthma and OSAS, focusing on the available evidence and looking for future research fields.

## 1. Introduction

Obesity in childhood represents a major global health challenge [1] mainly playing as a risk factor for morbidity in adulthood [2].

The prevalence of obesity shows a worrying increase of epidemic proportions. In the United States, 40% of adults and 19% of children have a condition of obesity [3].

The 2018 Global Nutrition Report showed that 40 million children below 5 years of age and 330 million patients aged 5–19 years were overweight or obese in 2016 [4]. Recent estimates suggest that in children and adolescents, the prevalence of obese girls rose from 0.7% in 1975 to 5.6% in 2016, and of obese boys from 0.9% in 1975 to 7.8% in 2016 [5]. 

Obesity is associated with several complications [6]. It is a major risk factor directly correlated with childhood hypertension and other metabolic chronic conditions including stroke [7], prediabetes and type 2 diabetes mellitus [8,9], dyslipidemia [10], non-alcoholic fatty liver disease, and/or gallstones [11,12].

Obesity alters osteo-cartilaginous structures leading to higher risk of forearm fractures following severe falls [13].

Obesity is a major preventable risk factor and disease modifier of many respiratory conditions, mainly involved in wheeze, dyspnea, and orthopnea both in adults and children, affecting the prevalence and the severity of several lung diseases, which has been also testified during the SARS-CoV-2 pandemics [14,15].

Obese children have an increased relative risk of 1.29 (95% CI: 1.16–1.42) for asthma and obesity induces 23% to 27% of asthma cases in children [16].

In a meta-analysis, Chen YC et al. reported that asthma incidence increases by a twofold risk in obese children, with higher susceptibility in males [17].

Moreover, obese children are at risk for developing obstructive sleep apnea syndrome (OSAS) and obesity hypoventilation syndrome. Aspiration pneumonia, pulmonary thromboembolism, chronic obstructive pulmonary disease (COPD), and respiratory failure may occur later in life [18,19,20].

Several mechanisms have been suggested to explain the association between obesity and respiratory symptoms, including reduced compliance of respiratory system, higher airway resistance, decreased lung volumes, and expiratory flow rates [21,22,23] and altered ventilation and gas exchange.

Authors looked for relevant studies published up to October 2020 on MEDLINE (PubMed). They searched through selected reviews, original articles, textbooks, and organizations’ websites. To achieve the highest sensitivity, a combination of keywords and indexed terms were used including as keywords “asthma” and “overweight” or “obesity” or “obstructive sleep apnea syndrome” and “children”. Relevant articles were included in this review with the aim of highlighting the most relevant connection between obesity and two frequent respiratory chronic diseases, asthma, and obstructive sleep apnea syndrome.

## 2. Obesity and Asthma

Asthma is an umbrella term that comprises heterogeneous conditions. Among the observable characteristics (phenotype) of asthma, those in obese children have been widely studied [24,25,26,27].

In children, asthma and obesity can co-occur [28]. Furthermore, asthma can predispose to obesity [29]. The risk of developing obesity is nearly twofold higher at age of eight in children who suffered from asthma at 3–4 years of age [30]. However, obesity can be a risk factor for asthma even in adolescence [31]. Lack of asthma control has a crucial role in the development of obesity. This is suggested by higher risk of obesity in adolescents with exercise-induced bronchospasm that limits physical activity [32,33].

Holguin et al. [34] identified two main “obese asthma” phenotypes among children and adolescents enrolled in the Severe Asthma Research Program (SARP), namely, the “early-onset obese-asthma” opposed to the “late-onset obese-asthma”.

Late-onset phenotype identified obese patients ≥12 years of age, who were older at the time of diagnosis, often female, with a higher prevalence of severe asthma and a poorer disease control (defined by more frequent exacerbations, hospitalizations and intensive care unit admissions), increased use of high-dose inhaled corticosteroids, oral corticosteroids, antileukotrienics and anti-IgE therapy, low Forced Expiratory Volume in the 1st second (FEV1), and Forced Vital Capacity (FVC) predicted values, and a neutrophilic/paucigranulocitic inflammation (T2 low).

The early-onset phenotype included patients <12 years of age, with a lower degree of female predominance and a similarity in asthma severity. Patients belonging to this subgroup required a greater use of health care resources (emergency visits and ICU admissions) and suffered from reduced quality of life, higher airway obstruction with marginally higher maximal FEV1 reversal and increased bronchial hyperresponsiveness. They also showed high IgE levels and positivity for skin prick test (T2 high). Both phenotypes lacked eosinophilia.

Other clinical characteristics of obese asthmatics have been found. Obese asthmatics have more severe symptoms than non-obese asthmatics [35], greater use of oral corticosteroids, a marker for asthma exacerbations [36], a reduced response to bronchodilator and to inhaled corticosteroids [37], perhaps explained by underlying non-eosinophilic mechanism and/or steroid resistance [38].

This is even more evident in asthmatic obese black and Latino adolescents [39] who have a bronchodilator unresponsiveness and a reduced control of symptoms with an increased need for oral corticosteroids and moderate-to-severe exacerbations.

Childhood obesity is responsible for a more frequent emergency-department utilization for asthma exacerbations [40] and overweight increases the likelihood of subsequent visits in Emergency Department (ED) in children admitted for asthma [41].

Among children hospitalized for acute asthma, obesity is associated with longer length of stay and higher risk of mechanical ventilation [42].

### 2.1. Obesity and Asthma: Pathogenesis

Co-occurrence of asthma and obesity may be due to common pathogenetic factors (Table 1).

#### 2.1.1. Environmental Factors

Common pathogenetic environmental factors include exposure to air pollutants [43,44,45], tobacco smoking both in utero and in children [46], Western diet [47] with high consumption of processed foods and saturated fatty acids [48], and low levels of Vitamin D [28].

#### 2.1.2. Genetics

Asthma and obesity share some genetic factors. Some genes involved in both asthma and in the increase of Body Mass Index (BMI) have been identified such as *PRKCA*, *LEP*, and *ADRB3* [49,50].

An interesting study conducted by Wang et al. identified common single nucleotide polymorphisms.

(SNPs) at 17q21 (an asthma-associated locus) associated with higher BMI only among asthmatic patients in two independent cohorts [51].

Rastogi et al. described differential patterns of DNA methylation in children as a function of their obesity and asthma status. In particular, they studied DNA methylation in peripheral blood mononuclear cells of eight asthmatic and obese children in comparison with a population of eight children with only asthma, obesity, or healthy controls. Children with both asthma and obesity had a distinctive appearance characterized by reduced methylation of the promoter of chemokine ligand type 5 (*CCL5*), interleukin 2 receptor antagonist (*IL2RA*), and T-box transcription factor 21 (*TBX21*) genes involved in Th1 polarization that is observed in obese asthmatics [52].

Hallstrand et al. studied genetic pleiotropy between asthma and obesity in two populations of same-sex twins; it was highlighted that asthma and obesity have an important genetic basis and that 8% of the genetic component of obesity is also present in asthma. The overlap of genetic causes associated with the two diseases seems to be explained by the polygenic etiology both of obesity and asthma and by the involvement of some common pathways (for example β2-adrenergic receptors and TNF-α level) [53].

Ahangari et al. found on mice and human subjects the expression of the Chitinase 3-like-1 (*CHI3L1*) gene inducible from a hyperlipidic diet and also related to the development of asthmatic disease. In particular, a case-control study has demonstrated that a hyperlipidic diet and exposure to aeroallergens increase the expression of the pulmonary Chitinase 3-like-1 (*CHI3L1*) gene, an evolutionarily conserved moiety involved in Th2 response. In addition, higher levels of *CHI3L1* were found in serum both in patients with increased visceral adiposity and in patients with persistent asthma [54].

#### 2.1.3. Lung Growth

Recently, attention has been increasing about dysanapsis, a phenomenon characterized by a mismatch between the increased degree of lung parenchymal development relative to airway caliber, leading to higher FEV1 and FVC though with a more evident effect on FVC. In 2005 European Respiratory Society defined dysanapsis as an airflow obstruction (testified by a reduction of FEV1/FVC) associated to normal FEV1 levels [55].

Studies on pediatric patients, despite few, appear to confirm the hypotheses that dysanapsis may play a role in airflow reduction in asthmatic obese.

Jones et al. recruited almost 188 children suffering from asthma (61%) or overweight/obese (38%) finding an independent association of both conditions with a dysanaptic growth of airways, influenced more by overweight than asthma [56].

Forno et al. conducted a huge study on more than 4000 children. Authors found a proportional increase of dysanapsis to BMI level and, interestingly, the presence of obesity-related dysanapsis lead to a more difficult control of symptoms and use of systemic corticosteroids in asthmatic children, leading to hypothesize that dysanapsis itself may lead to a reduced airway response to medications. Nevertheless, obese children in this cohort presented dysanapsis even in the absence of asthma features [57].

Dysanapsis appears as an interesting pathogenic feature of asthma, especially in overweight children, whose role still needs to be further clarified.

In obese subjects who develop asthma, obesity may be the cause of airway disease. Whereas in asthmatics who become obese, the overweight could influence asthma severity as comorbidity. Strunk et al. reported a worsening of airway obstruction in non-obese asthmatic children who later became obese. They experienced a significant decrease in FEV1/FVC and FEV1 and an inverse association between reduction of FEV1 percent predicted of 0.29 for each unit increase of BMI [58]. This may indicate that developing obesity in early adulthood could lead to a progressive airway obstruction.

#### 2.1.4. Lung Function

Obesity has been described to be detrimental to lung function. Obesity effects on lung function are different between adults and children.

Recent results indicate a more pronounced decline of Total lung capacity (TLC) in obese adults compared to children with an inverse association between BMI and lung volumes resulting in a restrictive ventilatory deficit.

Similarly, obesity was also associated with lower Residual volume (RV) among asthmatic and non-asthmatic adults [55,59,60,61,62,63].

At variance, obesity in children is associated with normal/increased FEV1 and FVC and low FEV1/FVC and forced expiratory flow at 25–75% of FVC (FEF25–75%). This pattern indicates either an obstructive lung deficit or airway dysanapsis [57,64] although a restrictive condition has also been rarely reported in children [65,66,67].

In obese subjects the impairment of the elastic properties of the chest results in a reduction in Functional Residual Capacity (FRC) and in an increase airway resistance. In particular, the variation of the parenchymal retractive forces on the airways results in low-volume breathing and a reduction in FRC leads to smooth muscle discharge that over-responds to broncho-obstructive stimuli in the obese non-asthmatic adult but not in asthmatics [68]. This finding underlines the crucial role of obesity in impairing lung function in otherwise healthy individuals.

Further studies are needed to evaluate the impact of obesity on the variation of RV and FRC in children, as the data available to date are insufficient.

Breathing at reduced lung volumes also increases airway hyperresponsiveness (AHR) which seems to have a direct correlation with the worsening of BMI [15,69].

Gender has an important modifier effect since obese girls tend to be more affected by onset of AHR than obese boys [70].

#### 2.1.5. Mechanical and Inflammatory Mechanisms

Both mechanical (reduced expansion of chest) and inflammatory mechanisms (enhanced release of inflammatory cytokines by adipose tissue) may be involved in lung function decrease and in asthmatic symptoms [23].

Regarding obesity-induced mechanical lung compression, abdominal adiposity can heighten diaphragm and diminish chest wall compliance reducing FRC and amplifying symptoms related to reduced airway caliber such as AHR, dyspnea, and wheeze. In agreement, in asthmatic adolescents, especially females, indicators of excess abdominal adiposity, such as high values of waist circumference and waist-to-height ratio, were observed [71].

Adipose tissue produces proinflammatory cytokines (leptin), while there is a reduced release of anti-inflammatory adipokines, including adiponectin.

Sideleva and colleagues recruited women undergoing bariatric surgery with a history of adult-onset asthma comparing them to women undergoing bariatric surgery with no evidence of asthma; subcutaneous fat, bronchoalveolar lavage (BAL) and brush biopsy were collected from each patient and were studied for inflammatory markers, including adipokines and cytokines. Inflammatory markers, especially in visceral adipose tissue, were found to be higher in patients with asthma, suggesting a central pathogenic role for visceral adipose tissue producing high levels of adipokines, able to affect airway reactivity [72].

In asthmatics, leptin increases adipose tissue macrophage release [73] of IL6, and TNF-alpha [74] and also induces a neutrophilic bronchial inflammation [75].

Obesity is associated with increased neutrophils both in the circulation and adipose tissue vasculature. Higher neutrophilic airway inflammation has been linked with higher CRP and IL-6, lower testosterone and no oral contraceptive pill use [76]. Increased circulant IL-6 levels are associated with more severe asthmatic symptoms [77].

While immunologic features of childhood asthma are characterized by a white blood cells (WBC) polarization towards T-Helper 2 (TH2) cells, obese children with asthma have a polarization towards T-Helper 1 (TH1) cells and increased activation of circulating monocytes, suggesting obesity-associated asthma as a distinct nosologic entity compared to obesity-unrelated asthma [78]. Furthermore, also insulin resistance seems to play a pivotal role between the TH1-polarization and lower lung volumes on Pulmonary Function Tests (PFTs) [22].

An experimental model hypothesized a role for Surfactant Protein-A (SP-A), a crucial mediator in reducing tissue and lavage fluid eosinophilia in allergic mouse models. Obese asthmatic patients expressed significantly lower levels of this protein in bronchoalveolar lavage (BAL) compared to non-obese asthmatics and non-obese non-asthmatic patients. Authors suggested that in obese patients high local levels of TNF-α could reduce SP-A secretion, leading to the eosinophilic asthma phenotype in a subgroup of obese asthmatic subjects [79].

#### 2.1.6. Oxidative Stress

Oxidative stress is an important feature of asthma in childhood [80]. Increased oxidative stress in the airways of obese asthmatic children has also been widely hypothesized as a potential link between asthma and obesity, even if no convincing evidence was found yet [22,81].

However, increased levels of oxidative stress have been found in obese patients with late-onset asthma and they have been associated with airway remodeling, impaired lung function and corticosteroid resistance. Nitric oxide synthase uncoupling, caused by dysfunction in the L-arginine/nitric oxide metabolism, and mitochondrial dysfunction could be two possible explanations of increased oxidative stress in obese asthmatics [82].

#### 2.1.7. Microbiome

A recent field of interest is the possible role of microbiome in obesity–asthma association. This finding may have several potential therapeutic implications considering the already proven effectiveness of probiotics in various diseases [83]. Modifications of the resident microbiome, with release of protective metabolites, including unsaturated fatty acids, tryptophan, or polysaccharides may prevent atopic diseases [47]. Obesity causes an alteration of the gut microbiota through a reduction in the ratio of Firmicutes to Bacteroidetes, leading to a changes of the metabolome potentially contributing to asthma development. This may be due to a pleiotropic effect on insulin sensitivity, host metabolism, changes in immune system, and changes in lung microenvironment [84,85].

The association between the gut microbiota and obesity-related asthma is supported by the evidence of microbiota influence on immune response via changes in IL-17A, an interleukin released by Th-17 cells crucial in the neutrophilic recruitment. It has been already demonstrated that germ free mice and those treated with antibiotics, have lower levels of intestinal Th-17 cells; the reverse phenomenon occurs in germ free mice colonized with filamentous bacteria [86,87,88].

Kim HY et al. demonstrated that wild-type mice on high-fat diet developed greater AHR compared with WT mice on control chow. On the contrary, this innate AHR was absent in IL-17A -/- mice both on high-fat diet and on a controlled diet [89].

Th-17 cells are implicated in neutrophilic asthma and are increased in obese subjects; high levels of Th17 cells were found in the sputum of obese asthmatic subjects [84]. In conclusion, high-fat diet could influence gut microbiota balance in obese subjects, increasing IL17A expression, being involved in neutrophilic obesity-related asthma.

### 2.2. Treatment

The role of obesity in asthma has also been underlined by studies on the effect of decreased body weight on asthma. After bariatric surgery, asthmatic obese adults undergo a significant improvement in asthma control, asthma quality of life, airways responsiveness to methacholine, and lung function [90].

In children, weight reduction may lead to an improved asthma control. The reduction of weight can be mainly achieved by dietary advice and increased physical activity. In children dietary intervention has been shown to be effective in improving lung function and asthma control but not systemic and airway inflammation [91].

Van Leeuwen et al. showed that weight loss was associated with improvement in exercise-induced bronchoconstriction and quality of life [92]. However, Willeboordse et al. [93] showed that reduction of BMI did not significantly affected lung function, asthma control, and quality of life suggesting that obesity has a pathogenetic role in a subset of asthmatics.

Furthermore, overweight and obesity appear to potentially mislead the physician in the management of asthma. The strong relationship between asthma and gastro-esophageal reflux, a very common symptom in overweight/obese patients, affects the perception of symptoms by patients even in presence of satisfactory lung function. This leads to unnecessary use of emergency therapy (short-acting beta agonists) potentially producing a vicious cycle where SABA itself worsens the gastro-esophageal reflux which, in turn, worsens the personal perception of symptoms by patients [94]. The treatment of obese asthmatics may, consequently, represent a real challenge.

The evidence that these patients present a higher percentage of neutrophils both in sputum and in blood, if compared to non-obese asthmatics, may nevertheless be helpful [95].

Though still not justified in all cases of asthma, some evidences support the use of macrolides in difficult-to-control neutrophilic asthma [96]. Macrolides may, in conclusion, represent a useful tool, whose use needs to be assessed on a case-by-case basis.

## 3. Obesity and OSAS

Another important pediatric respiratory condition associated with overweight and obesity is Sleep-Disordered Breathing (SDB), especially Obstructive Sleep Apnea Syndrome (OSAS). OSAS is a recurrent, partial, or complete, obstruction of the upper airways during sleep, resulting in disruption of normal gas exchange and sleep fragmentation. This association has been widely demonstrated in both adults [97] and children [98,99,100,101]. Despite this, over time, data about SDB and obesity have been conflictual. Verhulst et al. reported a prevalence of OSAS of 13–59% in obese children, compared to a prevalence of 1–2% in regular-weight children [102]. They also found an increased airway inflammation only in overweight children suffering from SDB [103]. In a later review, Kohler et al. highlighted a number of methodological limitations in previous studies on this topic, suggesting the association between overweight and SDB to be an epiphenomenon or even a result of chance. The authors then proposed the age and the ethnicity as two important factors influencing the SDB-overweight association, other than individual characteristics [104].

Hence, they showed that the strength of the association seems to proportionally increase with age [105], in particular for patients over 12 years of age, suggesting that the reason for this correlation may be a decreased upper airway tone in older children, leading to an easier collapse of airways.

A case control study was conducted to demonstrate that the risk of SDB was higher in obese children compared to normal population, and to understand the role of pharyngeal lymphoid tissue hypertrophy. Forty-six obese children (mean age 10.8 years) were compared with 44 healthy, normal weight children of the same age. A total of 34.8% of obese children reported snoring habitually more than 4 nights a week, compared to 15.9% of healthy controls. It was found that one-third of obese children had SDB and that both obesity and tonsillar hypertrophy were independent risk factors for the development of SDB [106].

In addition, pediatric OSAS, like obesity, has been associated with increased cardiovascular morbidity [100]. This correlation has been attributed to a persistence of the sympathetic tone, both during the day and the night. Systolic blood pressure is higher in pediatric patients suffering from SDB than in controls matched by age. It could depend on hypoxia-induced stimulation of sympathetic tone, increased catecholamine production by adrenal glands and dysfunction of renin–angiotensin–aldosterone pathway. Inflamed microvasculature may contribute to the pathogenesis but its role needs to be properly evaluated [107].

### 3.1. Obesity and OSAS: Diagnosis

The American Academy of Sleep Medicine (AASM) Manual for Scoring of Sleep and Associated Events recommends monitoring hypoventilation through diagnostic polysomnograms in children with suspected OSAS [108].

Standard pediatric polysomnography offers a wide perspective on patient’s sleep patterns by recording multiple physiologic signals consisting of electroencephalogram, electrocardiogram, electromyogram, electro-oculogram, oro-nasal airflow, respiratory effort, and peripheral oxygen saturation.

In 2002 the American Academy of Pediatrics prepared a technical report on the best diagnostic tests for sleep-disordered breathing; 2115 works were revised. This review concluded that overnight polysomnography (PSG) was the reference method for the diagnosis of OSAS, also in obese kids. On the other hand, they studied pulse oximetry’s effectiveness; it resulted specific but not sensitive enough [109].

Nixon GM et al. tried to develop a scoring system in which pulse oximetry proved a diagnostic inferiority when compared to PSG [110]. Similar findings were found in subsequent studies and pulse oximetry alone is currently considered to be insufficient for the diagnosis of OSAS [111], especially in obese subjects.

Paruthi et al. analyzed the end-tidal Carbon Dioxide (EtCO_2_) Measurement during Pediatric Polysomnography. Their findings suggested that the levels of EtCO_2_ may contribute to the diagnosis similarly to the polysomnographic indices [112].

Transcutaneous monitoring of CO_2_ (TcCO_2_) appears to be a promising diagnostic tool in obese patients, showing a good correlation with arterial blood PaCO_2_ levels [113]. TcCO_2_ changes has recently been evaluated also in children showing a good correlation with airways obstruction in subjects suspected of sleep disordered breathing [114] and in children undergoing tonsillectomy [115].

### 3.2. Obesity and OSAS: A Bidirectional Causality

The interaction between OSAS and obesity is very complex, and their coexistence may negatively influence the severity of both conditions, creating an interleaved vicious cycles. Systemic inflammation, oxidative stress, metabolic affection, and gut microbiota have a synergistic role in determining the pathogenesis of these two diseases [116].

Obesity is mainly involved in the pathogenesis of OSAS by various mechanisms but at the same time, OSAS can cause obesity due to several factors such as reduced physical activity, insulin resistance and elevated ghrelin levels [117,118,119]. Obesity is responsible of adipose deposits in upper airway lumen and muscles, causing their collapse especially during sleep, predisposing to the development of OSAS [120].

Furthermore, the abdominal adipose tissue is responsible for a lower diaphragmatic excursion and a reduction of the intrathoracic volume, especially in the supine position, increasing the respiratory work during sleep [121].

Leptin is considered one of the main explanations of the association between obesity and OSAS, since obese subjects show resistance to leptin function. This hormone would act on central chemoreceptors causing an increase in triggering ventilation, but obese patients show a failure in this mechanism because of a resistance to leptin’s action [120].

Leptin is a peptide hormone produced primarily in adipose tissue, that also influences energy homeostasis, metabolism, inflammation, and sympathetic nerve activity. Recent studies reported a positive correlation between blood leptin levels and OSAS severity [122,123].

Obesity is associated to an impaired adipose tissue function, which leads to adipocyte hypertrophy, hypoxia and activation of inflammatory processes within adipose tissue [124]. In addition, the intermittent hypoxia associated with OSAS is responsible for the production of inflammatory cytokines in adipose tissue [125].

A Chinese case-control study from 2016 explored the possible pathogenic role for adipokines on a small sample of OSAS adult patients. All patients underwent PSG monitoring, and a venous blood sample was collected before and after PSG to test a defined group of adipokines (chemerin, macrophage migratory inhibitory factor, visceral adipose tissue-derived serine protease inhibitor, and chemokine CXCL5). Multiple regression analyses showed that altered BMI and Apnea Hypopnea Index (AHI) were associated with high plasmatic levels of these adipokines in OSAS patients, finding a significant correlation between specific adipokines, OSAS severity and obesity [126].

Many studies confirm that obesity and OSAS share similar dysregulation in metabolic process. Indeed, both conditions are characterized by oxidative stress, which influences their development and progression [127,128].

In particular, intermittent hypoxia-reoxygenation in OSAS patients is responsible for ATP depletion and xanthine oxidase activation, with consequent production of oxygen-derived free radicals [129] causing an inflammatory status [130].

At the same time, oxidative stress and inflammation in obesity enhance OSAS.

Moreover, an inflammatory state in obese children suffering from OSAS is attested by the upregulation of pro-inflammatory signaling pathways (NFkB, HIF-1a, and adipokines) which lead to greater expression of pro-atherogenic factors and endothelial dysfunctions [131].

Promising data were found about the relationship between platelet activation and OSAS, with many authors hypothesizing platelet activation’s pathogenic role to be central in OSAS.

Kurt OK et al. studied the correlation of some blood parameters, including Mean platelet volume (MPV) and platelet distribution width (PDW) to OSAS severity. They found an elevation of PDW in severe OSAS, suggesting a role for platelet activation as a marker of OSAS severity. Included patients presented mean BMI levels ranging from 28.4 (overweight) to 33.2 (obese) [132].

In 2015, Akyol S et al. confirmed that MPV was associated with OSAS severity but also independently linked to high sensitivity C-reactive protein, a marker of systemic inflammation [133].

A recent meta-analysis showed higher MPV values in pediatric patients suffering from SDB than in healthy children, revealing an increased platelet activity. They also demonstrated a reduction of the parameter after adenoidectomy and adenotonsillectomy [134].

In recent years, human intestinal microbiota has emerged as a key factor for the development of both obesity and OSAS [135,136]. Many studies also confirm the close relationship between lungs and gut as a part of the mucosal immune system: an inflammatory status in one of these organs influences each other’s homeostasis [137,138].

A high-fat diet might be responsible for a dysbiosis in the gut microbiota that could have a crucial role in the development of a low-grade inflammatory status [139,140].

Additionally, in OSAS, intermittent hypoxia and sleep deprivation further influence gut microbiota leading to a systemic inflammatory process which contributes to cardiovascular and metabolic morbidities [136].

It is assumed that the so called mutual “organ crosstalk” between respiratory system, adipose tissue and intestine can also explain the complex relationship between OSAS and obesity.

Among the therapeutic approaches to treat OSAS, the weight loss and lifestyle changes are the most effective [141]. Medical and surgical approach [142,143] are valid solutions for treatment. Gileles-Hillel A et. al proposed continuous positive airway pressure (CPAP) therapy in adults and adenotonsillectomy in children to treat OSAS but the benefit on metabolic function remains doubtful [144].

Confirming the strict interdependence between the two pathologies, many studies observed that the resolution of OSAS following adenotonsillectomy leads to improvement in metabolic disorders related to obesity such as dyslipidemia. OSAS resolution is associated to low-density lipoprotein (LDL) and apolipoprotein B (ApoB) decrease and to a high-density lipoprotein (HDL) increase [131].

An Australian study analyzed polysomnographic results before and after bariatric surgery demonstrating an important decrease in the severity of OSAS after weight reduction [145].

Andersen IG et al. conducted a prospective longitudinal study on 62 children and adolescents with BMI > 90th percentile with OSAS (defined by AHI > 2) who underwent sleep examinations and anthropometric assessments at baseline and after 6 and 12 months from baseline; they were subjected to an individualized obesity treatment regarding lifestyle changes.

They observed a normalization of AHI (apnea-hypopnea index) in 38% of children after about 6 months of obesity treatment and in 44% one year after starting treatment. They also assessed that children with tonsillar hypertrophy achieved same effect as children without tonsillar hypertrophy [146,147]. (Table 2).

## 4. Obesity and COVID 19

Recent data on the spread of the new COVID 19 virus in the pediatric population seem to demonstrate the greater susceptibility of obese children to the development of a more severe respiratory disease than their normal-weight peers. Clinically, COVID 19 infection in pediatric age is lesser severe than in the adult population and occurs mainly with cough and fever, sore throat, myalgia, rhinorrhea, nasal obstruction, diarrhea, and vomiting. Obesity seems to increase susceptibility to infection and seems to worsen the prognosis; during the epidemic in Canada and in New York obesity was the most frequent comorbidity in children admitted to the ICU for COVID 19 infection; this association has been found in several studies on the adult population. A link between obesity and severity of COVID 19 respiratory infection probably lies not only in the causes listed above that predispose obese child to respiratory disease (chronic inflammation, insulin resistance, mechanical factors, prothrombotic state, Vitamin D and other micronutrient deficiencies, impaired immunological response, and dysbiosis) also in the greater expression of the ACE2 receptor (which internalizes the virus in the cells of the respiratory system). The overexpression of the ACE2 receptor has been demonstrated in mouse models subjected to a hyperlipidemic diet and it is also present in obese subjects who take therapy with RAAS system inhibitors for cardiovascular comorbidities associated with obesity. However, we still have few data on the pediatric population and COVID 19 infection and further studies are needed [148].

## 5. Conclusions

Though not overwhelming, evidences are convincing about the key role played by obesity in asthma and OSAS.

Regarding the link between asthma and obesity, a gene-by-environment interaction should be thoroughly investigated. The discovery of modifiable environmental factors such as diet that can influence gene expression through epigenetic mechanisms inducing both obesity and asthma in predisposed subjects will be crucial to prevent the spread of these two conditions.

Although the contribution of obesity in patients with OSAS appears to be due to mechanical factors, evidences strongly support the key role of inflammatory cytokines with a pro-inflammatory signaling pathways, a reduced effectiveness of leptin action combined with high adipokine levels. The role of platelet activation in OSAS asthmatic patients appears to be crucial but yet to be fully explained. At last, gut dysbiosis significantly affects the lung-gut axis leading to an impairment of lung function.

Studies assessing the correlation between the pathogenic role of platelet activation and BMI levels may be useful for a deeper understanding of the issue, such as studies exploring the lung–gut axis in a detailed manner.

In order to prevent obesity and the consequent complications, family education, and precocious changes of the lifestyle are essential. We believe that a healthy diet would lead to an overall improvement of the quality of life in obese children suffering from respiratory diseases.

## Figures and Tables

**Table 1:** The association between obesity and asthma.

Obesity and Asthma
Environmental factors: Air pollutants and tobacco smoking Diet Low levels of Vitamin D Genetic factors *PRKCA*, *LEP* and *ADRB3* genes Nucleotide polymorphisms (SNPs) in 17q21 Reduced methylation of the promoter of *CCL5*, *IL2RA* and *TBX21* genes Gene inducible from a hyperlipidic diet (*CHI3L1*) Lung growth Dysanapsis Mechanical factors Impairment of the elastic properties of chest wall Increase airway resistance Lower RV Abdominal adiposity Microbiome Oxidative stress Immunological factors Polarization towards T-Helper 1 (TH1) cells Proinflammatory cytokines (leptin)

*PRKCA*: protein kinase C alpha; *LEP*: leptin; *ADRB3*: beta3-Adrenergic receptor; *CCL5*: chemokine ligand type 5; *IL2RA*: interleukin 2 receptor antagonist; *TBX21*: T-box transcription factor 21; *CHI3L1* Chitinase 3-like-1; RV residual volume.

**Table 2:** The association between obesity and Obstructive Sleep Apnea Syndrome.

Obesity and OSAS
**Mechanical adiposity factors** Upper airways collapse Lower diaphragmatic excursion Reduction of the intrathoracic volume Increased respiratory work during sleep **Inflammatory cytokines and hormones** Resistance to leptin action Activation of pro-inflammatory signaling pathways High adipokine levels and inflammatory markers High MPV High production of oxygen-derived free radicals Lung-Gut Axis

OSAS: Obstructive Sleep Apnea Syndrome; MPV: Mean Platelet Volume.
